# Genetic Overlap Between Dilated Cardiomyopathy and Neurological Disorders: Insights from a Next-Generation Sequencing Study

**DOI:** 10.3390/diagnostics16152395

**Published:** 2026-07-30

**Authors:** Maria Grazia Salluzzo, Francesca A. Schillaci, Elisa Zago, Sofia Fucile, Luca Marcolungo, Pietro Schinocca, Giuseppe Lanza, Raffaele Ferri, Giuseppe Leonardi, Michele Salemi

**Affiliations:** 1 Oasi Research Institute-IRCCS, 94018 Troina, Italy; msalluzzo@oasi.en.it (M.G.S.); faschillaci@oasi.en.it (F.A.S.); pschinocca@oasi.en.it (P.S.); glanza@oasi.en.it (G.L.); rferri@oasi.en.it (R.F.); 2 Personal Genomics S.r.l., 37136 Verona, Italy; elisa.zago@personalgenomics.it (E.Z.); sofia.fucile@personalgenomics.it (S.F.); luca.marcolungo@personalgenomics.it (L.M.); 3 Department of Surgery and Medical-Surgical Specialties, University of Catania, 95123 Catania, Italy; 4 Cardiology Unit, A.O.U. Policlinico Rodolico-San Marco, 95123 Catania, Italy; leonardi.pippo@gmail.com

**Keywords:** dilated cardiomyopathy, neurological diseases, next generation sequencing, TTN gene

## Abstract

**Background/Objectives**: Dilated cardiomyopathy (DCM) is a genetically heterogeneous myocardial disorder. Emerging evidence suggests that some genes implicated in DCM may also be associated with neurological disorders, supporting the concept of genetic pleiotropy. This study explored the intersection between cardiac and neurological genetics, with the aim of identifying candidate genes that may contribute to shared pathogenic pathways linking these clinically distinct conditions. **Methods**: We performed exome sequencing in 149 patients with echocardiographically confirmed DCM and subsequently applied an in silico filter to a predefined list of 211 genes associated with inherited cardiomyopathies. Variants were classified according to the American College of Medical Genetics and Genomics (ACMG) criteria. Genes with validated evidence for DCM according to the Clinical Genome Resource (ClinGen) were further investigated through the Human Gene Mutation Database (HGMD) and a focused literature review to identify reported associations with neurological disorders. **Results**: Genetic variants in DCM-associated genes were identified in 105 patients. Overall, 137 variants were detected, including pathogenic variants and variants of uncertain significance. The most frequently involved genes were *TTN*, *FLNC*, and *MYH6*. Several DCM-associated genes also showed reported associations with neurological disorders, including autism spectrum disorder, Alzheimer’s disease, Parkinson’s disease, epilepsy, and schizophrenia. Among them, *TTN*, *FLNC*, *RYR2*, and *SCN5A* displayed the broadest overlap between cardiac and neurological phenotypes. **Conclusions**: These descriptive findings show that variants were most frequently observed in *TTN*, *FLNC*, and *MYH6* and that several genes included in the ClinGen DCM curation framework have also been independently reported in neurological disorders. Because most identified variants were VUS and no control group or systematic neurological phenotyping was available, the findings indicate gene-level co-annotation only and do not establish variant enrichment, shared pathogenic mechanisms, or clinical overlap.

## 1. Introduction

Cardiomyopathies are a heterogeneous group of myocardial disorders characterized by structural and functional abnormalities of the heart muscle, often leading to mechanical dysfunction, electrical instability, or both. According to their predominant phenotype, cardiomyopathies are commonly classified as hypertrophic cardiomyopathy (HCM), restrictive cardiomyopathy (RCM), dilated cardiomyopathy (DCM), and arrhythmogenic cardiomyopathy (ACM) [1,2,3]. Among these conditions, DCM is one of the most common forms of non-ischemic cardiomyopathy and is defined by left ventricular, or biventricular, dilation associated with systolic dysfunction in the absence of abnormal loading conditions or significant coronary artery disease [1,2]. DCM represents a major cause of heart failure, arrhythmias, cardiac transplantation, and premature mortality worldwide [2]. The annual incidence in Europe and North America ranges from 5 to 7.9 cases per 100,000 people [4]. Other epidemiological studies have consistently reported a higher prevalence and greater severity of DCM in males than in females, with an estimated male-to-female ratio of approximately 2:1. The biological mechanisms underlying these sex-related differences remain incompletely understood and may involve genetic, epigenetic, hormonal, and environmental factors, as well as differences in disease penetrance and expressivity [5,6].

DCM can be classified into genetic, either familial or sporadic, and acquired forms. Secondary forms may result from ischemic injury, myocarditis, exposure to cardiotoxic agents, metabolic disorders, or endocrine abnormalities [1,2]. Over the last two decades, advances in next-generation sequencing (NGS) technologies have substantially improved our understanding of the genetic basis of DCM, revealing marked genetic heterogeneity and identifying numerous disease-associated genes [1,2]. Genetic testing is increasingly recognized as an essential component of clinical evaluation, as it can provide valuable information for diagnosis, prognosis, family screening, and personalized management strategies [7,8]. Among the genes most frequently implicated in DCM, TTN has attracted particular attention. This gene encodes titin, the largest known human protein and a critical structural component of the sarcomere. Multiple studies have shown that pathogenic TTN variants, particularly truncating variants, represent the most common genetic cause of DCM [9,10,11]. A meta-analysis published in 2019 estimated that TTN variants account for approximately 17% of DCM cases, underscoring their major contribution to disease pathogenesis [12].

Growing evidence also indicates a close relationship between cardiovascular and neurological diseases. Cardiomyopathies are frequently observed in patients with neuromuscular disorders, including muscular dystrophies, congenital myopathies, myofibrillar myopathies, and metabolic myopathies [13,14,15,16,17]. More recently, increasing attention has focused on the overlap between cardiovascular diseases and neurodegenerative disorders, which appear to share molecular pathways, biological mechanisms, and genetic risk factors [18,19]. A recent review by Cousineau and colleagues highlighted several biological links between cardiovascular diseases and neurodegenerative disorders, including Alzheimer’s disease (AD), Parkinson’s disease (PD), Huntington’s disease (HD), amyotrophic lateral sclerosis (ALS), and multiple sclerosis (MS) [18]. These interactions involve common pathways related to mitochondrial dysfunction, neuroinflammation, autonomic regulation, vascular impairment, and cellular stress responses. In addition, several genes associated with cardiovascular diseases have also been implicated in neurological disorders, suggesting the existence of shared pathogenic mechanisms [18].

Additional evidence supports the concept of a heart–brain genetic axis. Various forms of parkinsonism and PD have been associated with different cardiomyopathic phenotypes [20,21,22,23]. Transcriptomic analyses of PD brain tissue have identified dysregulated pathways related to cardiac muscle contraction and cardiomyopathy-associated genes [24]. Similarly, genome-wide expression studies have suggested shared molecular mechanisms between autism spectrum disorder (ASD) and DCM [25]. Moreover, mutations in the PSEN2 gene, originally described in familial AD, have also been reported in patients with Lewy body dementia, frontotemporal dementia, PD with dementia, and DCM [26,27]. Taken together, these observations support the hypothesis that several genes implicated in DCM may exert pleiotropic effects across different organ systems, including the central nervous system. Therefore, investigating the genetic overlap between DCM and neurological disorders may provide novel insights into shared biological pathways and disease mechanisms.

In the present study, we used NGS to identify genetic variants in patients with DCM and subsequently explored whether the involved genes had previously been associated with neurological disorders. By examining the intersection between cardiac and neurological genetics, we aimed to identify candidate genes that may contribute to common pathogenic pathways linking these apparently distinct clinical conditions.

## 2. Materials and Methods

### 2.1. Participants

This observational study included 149 consecutive patients with a diagnosis of DCM established by echocardiographic evaluation. The cohort comprised 100 males and 49 females, with a mean age of 52.57 ± 15.19 years. All participants were recruited at the Cardiology Unit of the Policlinico University Hospital “G. Rodolico–San Marco” of Catania, Italy. A comprehensive clinical assessment and review of medical history were performed for all participants. According to the available clinical records and routine medical history, none of the enrolled subjects had a documented diagnosis of a neurological disorder at recruitment. No standardized neurological, neuropsychological, or instrumental neurological assessment was performed; therefore, subclinical or unrecognized neurological manifestations could not be excluded.

Written informed consent was obtained from all participants before inclusion in the study. The study protocol was approved by the Ethics Committee of the Oasi Research Institute-IRCCS, Troina, Italy (approval code: CEL-IRCCS OASI/10-04-2025/01; approval date: 10 April 2025) and was conducted in accordance with the ethical principles of the Declaration of Helsinki and its subsequent amendments.

### 2.2. DNA Extraction

Peripheral blood samples were collected in EDTA tubes, and DNA extraction was performed according to the Lahiri and Nurnberger protocol [28].

Personal Genomics S.r.l., based in Verona (VR, Italy), performed quality control and next-generation sequencing (NGS) experiments on all samples. DNA concentration and purity were assessed using a NanoDropOne spectrophotometer (Thermo Fisher Scientific, Waltham, MA, USA), and sample quality was evaluated using a TapeStation 4200 system (Agilent Technologies, Santa Clara, CA, USA).

### 2.3. NGS Sequencing

Indexed libraries were prepared from 150 ng of purified DNA using the egSEQ Exome Inherit Panel (Edinburgh Genetics, Penicuik, UK), which provides comprehensive coverage of the whole exome.

Library quantification was performed using the 4200 TapeStation System (Agilent, Santa Clara, CA, USA) and the Qubit fluorometer (Thermo Fisher Scientific, Waltham, MA, USA). Libraries were then pooled in equimolar amounts, resulting in a final pool concentration of 2 nM. Sequencing and cluster generation were performed on the Novaseq X Plus platform (Illumina, San Diego, CA, USA) in a 2 × 150 paired-end format, achieving an average coverage depth of approximately 100×. Sequence files (.fastq files) underwent quality control analysis using FastQC v0.12.1 (http://www.bioinformatics.babraham.ac.uk/projects/fastqc, accessed on 1 January 2025). Paired-end read alignment and variant calling were performed using Dragen v3.9. Sequencing adapter trimming and quality filtering (removal of low-quality bases with a Phred quality score lower than 20 at the 3′ extremity) were performed internally by the Dragen pipeline prior to aligning the reads to the employed reference genome (GRCh38).

Alignment statistics and coverage metrics were calculated on the coding region of the 211 target genes associated with inherited cardiomyopathies (Supplementary Table S1). All the alignment and coverage metrics were evaluated. Given the high quality across all samples, evidenced by an average mean target coverage of 166×, more than 98% of target bases were covered with at least 20 reads, and more than 98% of genotypable bases were covered, no samples were excluded from further analysis. After exome sequencing, alignment, and variant calling, the resulting VCF files were filtered in silico to retain variants located within a predefined list of 211 genes associated with inherited cardiomyopathies.

### 2.4. Variant Filtering, Annotation and Data Analysis

Identified variants were filtered according to a minor allele frequency (MAF) < 1%, using the genomic reference datasets 1000 Genome Project PHASE3, ExAC 0.3.1 (Exome Aggregation Consortium), gnomAD 2.0.1 (Genome Aggregation Database), and ESP/EVS ESP6500SI-V2 (NHLBI Exome Sequencing Project). The potential functional impact of each variant was assessed using multiple in silico prediction tools, including SIFT, PolyPhen-2, and REVEL. Additional annotation and interpretation were performed using eVai enGenome (https://evai.engenome.com/#login, accessed on 1 January 2025) and Franklin via QIAGEN (https://franklin.genoox.com/clinical-db/home, accessed on 1 January 2025), using Variant Call Format (.vcf) files as input.

Variant classification was carried out according to the recommendations of the American College of Medical Genetics and Genomics (ACMG), integrating available clinical, genetic, and bioinformatic evidence. To specifically investigate variants associated with DCM, we subsequently focused on the genes included in the Clinical Genome Resource (ClinGen) framework for dilated cardiomyopathy (Table 1). These genes were selected according to the strength of currently available evidence supporting their association with DCM.

To explore potential links between DCM and neurological disorders, all ClinGen-classified DCM genes were further investigated using the Human Gene Mutation Database (HGMD) and an extensive literature review. This approach allowed us to identify genes potentially implicated in both cardiac and neurological phenotypes and to assess the extent of genetic overlap between these conditions.

### 2.5. Statistical Analysis

Descriptive statistics were used to summarize the demographic and genetic characteristics of the study cohort. Continuous variables are presented as mean ± standard deviation (SD), whereas categorical variables are expressed as absolute frequencies and percentages. Genetic findings were analyzed both at the patient level, including the number and proportion of subjects carrying variants in DCM-associated genes, and at the variant level, including the total number of variants, affected genes, and ACMG classification categories.

For comparative analyses, variants classified as Likely Pathogenic and Pathogenic were combined into a single LINK.PATH+PATH category and compared with variants classified as VUS. Sex-related differences in the proportion of patients carrying variants in ClinGen-classified DCM genes and in the distribution of variant categories were evaluated using the chi-square test or Fisher’s exact test, as appropriate according to expected cell counts. Odds ratios (ORs) with 95% confidence intervals (CIs) were calculated when applicable. Statistical significance was set at *p* < 0.05, and all tests were two-tailed.

Given the exploratory nature of the study, no correction for multiple comparisons was applied. The statistical analysis was primarily intended to describe the distribution of identified variants in the cohort and to assess potential sex-related differences in variant frequencies. The analysis of the overlap between DCM-associated genes and neurological disorders was descriptive and based on gene-level associations derived from HGMD and literature review; therefore, no causal or phenotype-level inference was performed. All statistical analyses were performed using standard statistical software.

## 3. Results

### 3.1. Identification of Variants Associated with DCM

After application of the filtering criteria described in the Section 2.4, no relevant genetic variants were identified in 11 of the 149 enrolled subjects. In an additional 33 patients, variants were detected only in genes not currently classified as causative or definitively associated with DCM according to the ClinGen framework (Table 1). Accordingly, 105 patients, including 72 males and 33 females, with a mean age of 52.73 ± 14.62 years, were found to carry variants in genes classified as associated with DCM. Overall, variants were identified in 30 of the 64 ClinGen-listed DCM genes, corresponding to approximately 47% of the genes included in this reference framework (Supplementary Table S2).

**Figure 1:**
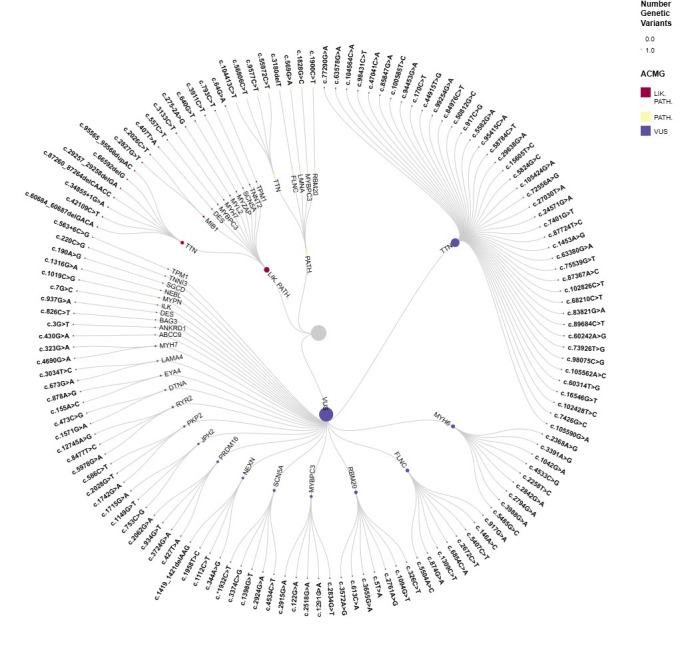
A circular dendrogram illustrating the data from Supplementary Tables S2 and S3, highlighting the classification of variants identified in the 105 cases in our cohort, the genes involved and considered implicated in DCM, as well as the identified variants. In the figure, the ACMG classification legend is represented by three colors. The circles gradually increase in size (from the outside in) because they represent the sum of each individual dot and are therefore directly proportional to the gene’s involvement and the ACMG classification. The figure was created using RAWGraphs 2.0 (DensityDesign, Calibro, and Inmagik, Apache License 2.0) (https://app.rawgraphs.io/, accessed on 1 January 2025).

A total of 137 genetic variants were detected in the study cohort (Figure 1 and Supplementary Table S3). Of these, 112 were classified as Variants of Uncertain Significance (VUS), 17 as Likely Pathogenic, and 8 as Pathogenic according to ACMG criteria. No significant sex-related differences were observed in the distribution of genetic variants (Figure 1 and Supplementary Table S2).

The genes most frequently affected by genetic variants were *TTN*, *FLNC*, and *MYH6*. *TTN* showed the highest number of Pathogenic or Likely Pathogenic variants, accounting for 11 of the variants in these categories. In addition, *TTN* was also the gene with the highest number of VUS, with 53 variants identified. Taken together, these findings indicate that *TTN* was the most frequently altered gene in our DCM cohort. *FLNC* was the second most frequently involved gene, with eight VUS and one Pathogenic variant identified in the study population. Variants in *MYH6* were also recurrently observed, further supporting the relevance of this gene in the genetic architecture of DCM (Figure 1 and Supplementary Table S3).

### 3.2. DCM-Associated Genes and Their Relationship with Neurological Disorders

**Figure 2:**
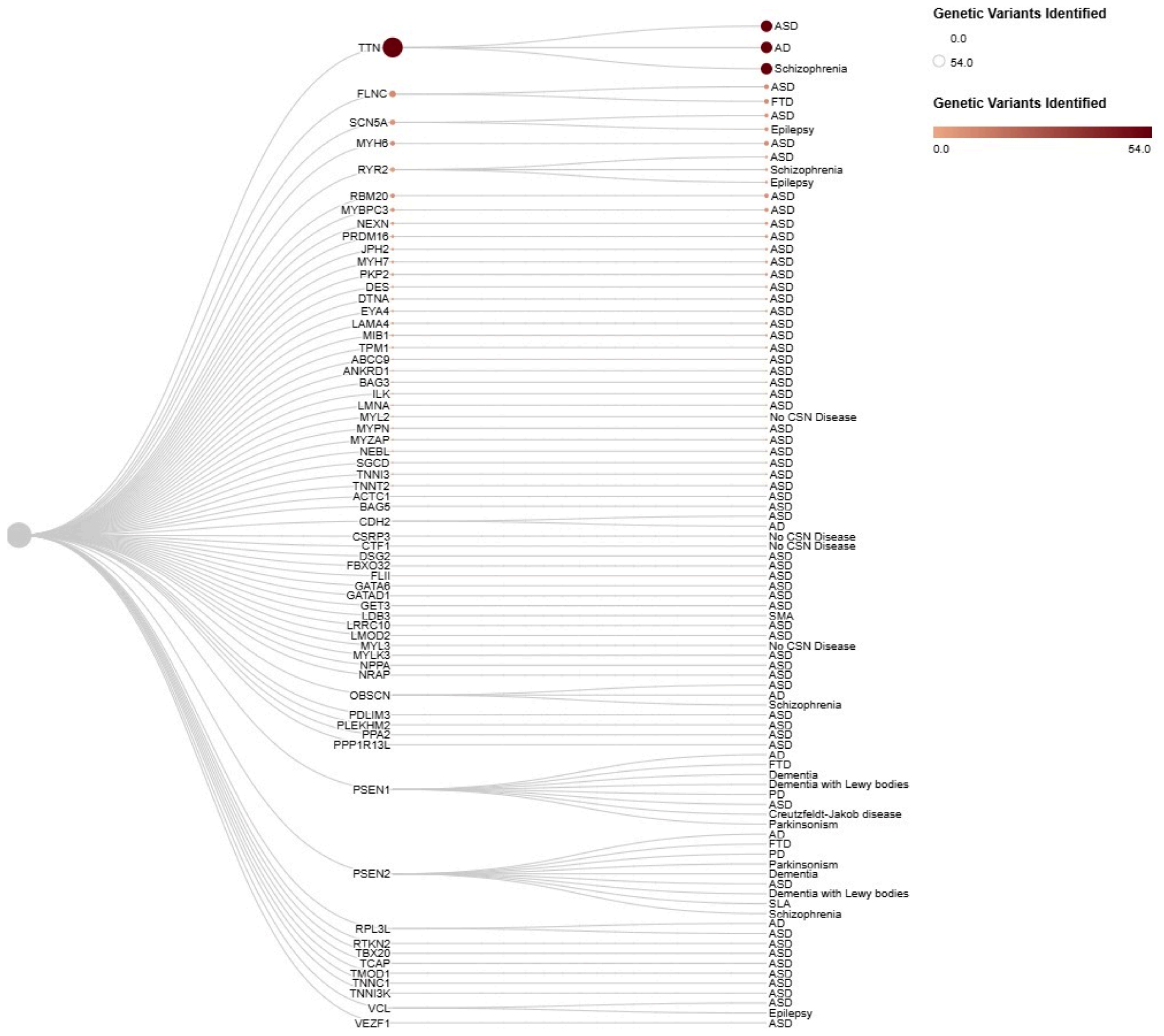
A linear dendrogram depicting the data from Supplementary Table S3, showing the involvement of various genes, previously implicated in DCM, in different CNSDs. In the figure, the legend shows a color gradient that indicates the number of variants identified (from 0 to 54), as well as the size of the dot. For example, 54 variants were detected for the TTN gene, which is indicated by the size of the dot and its color. The spheres on the left, labeled with the genes, are proportional in size to the sum of the spheres on the right. Genes in which no variants were detected have neither spheres nor color, but these are genes for which associations with CNSD (multiple and single branches) have been identified via HGMD. The figure was created using RAWGraphs 2.0 (DensityDesign, Calibro, and Inmagik, Apache License 2.0) (https://app.rawgraphs.io/, accessed on 1 January 2025).

The literature review and HGMD-based analysis showed that several genes associated with DCM have also been reported in neurological disorders. In particular, gene-level overlap was observed with genes previously associated with autism spectrum disorder (ASD), Alzheimer’s disease (AD), Parkinson’s disease (PD), frontotemporal dementia (FTD), epilepsy, schizophrenia, and other central nervous system disorders (Figure 2 and Supplementary Table S3).

Among the identified genes, *TTN*, *FLNC*, *RYR2*, and *SCN5A* showed the broadest spectrum of reported associations with neurological disorders (Figure 2 and Supplementary Table S3). Notably, *TTN* and *FLNC* were also among the genes most frequently affected by variants in our DCM cohort. Figure 2 illustrates the overlap between genes implicated in DCM and those previously reported in neurological disorders, highlighting the potential pleiotropic role of selected genes involved in both cardiac and nervous system function.

### 3.3. Distribution of DCM-Associated Genetic Variants

The proportion of patients carrying variants in ClinGen-classified DCM-associated genes was 70.5% overall, corresponding to 105 of the 149 enrolled subjects. No relevant variants were detected in 11 patients (7.4%), whereas 33 patients (22.1%) carried variants only in genes not currently classified as causative or definitively associated with DCM according to the ClinGen framework. The frequency of DCM-associated variant carriers did not differ significantly between males and females: 72 of 100 males (72.0%) and 33 of 49 females (67.3%) carried variants in ClinGen-classified DCM genes (χ^2^ = 0.34, *p* = 0.559; Fisher’s exact test *p* = 0.571; OR = 1.25, 95% CI: 0.60–2.61).

At the variant level, the 137 identified variants corresponded to a mean of 1.30 variants per carrier and 0.92 variants per enrolled subject. Variants involved 30 of the 64 ClinGen-listed DCM genes, corresponding to 46.9% of the reference gene set. According to ACMG classification, 112 variants were VUS (81.8%), 17 were Likely Pathogenic (12.4%), and 8 were Pathogenic (5.8%). When Likely Pathogenic and Pathogenic variants were combined, the LINK.PATH+PATH category accounted for 25 of 137 variants (18.2%). TTN accounted for 64 of all identified variants (46.7%), including 53 of 112 VUS (47.3%) and 11 of 25 LINK.PATH+PATH variants (44.0%).

## 4. Discussion

### 4.1. Main Findings

In this observational NGS study, variants in genes included in the ClinGen DCM curation framework were identified in 105 of 149 patients. *TTN*, *FLNC*, and *MYH6* were the genes containing the largest numbers of identified variants. However, because most variants were classified as VUS, their numerical frequency should not be interpreted as evidence of pathogenicity or as confirming a causal contribution to DCM. In addition, our literature- and database-based analysis showed that several genes implicated in DCM have also been reported in neurological disorders, including ASD, AD, PD, epilepsy, schizophrenia, and other central nervous system disorders. These findings support the existence of a potential gene-level overlap between cardiovascular and neurological diseases.

DCM is characterized by marked genetic heterogeneity, and more than 60 genes have been definitively or strongly associated with the disease [30,31]. The increasing availability of high-throughput sequencing technologies has substantially improved our understanding of the molecular basis of DCM, enabling the identification of disease-causing variants and facilitating more personalized approaches to diagnosis, prognosis, and family screening.

One of the most relevant findings of our study was the high frequency of variants affecting FLNC. FLNC encodes filamin-C, an actin-binding protein highly expressed in striated muscle and predominantly localized within the Z-disc and intercalated discs, where it contributes to sarcomeric integrity, mechanotransduction, and myofibrillar repair [32,33]. Pathogenic *FLNC* variants have emerged as an important cause of inherited cardiomyopathies, particularly arrhythmogenic and dilated phenotypes, mainly through haploinsufficiency mechanisms resulting in loss of functional protein [34,35]. Clinically, FLNC-related cardiomyopathy is frequently associated with ventricular dysfunction, myocardial fibrosis, ventricular arrhythmias, and increased risk of sudden cardiac death [36,37]. The relatively high number of *FLNC* variants identified in our cohort is therefore consistent with current knowledge regarding the pathogenic relevance of this gene in DCM.

Interestingly, *FLNC* has also been reported in studies investigating ASD and other neurodevelopmental disorders [38,39]. Although the biological significance of these observations remains incompletely understood, they suggest that *FLNC* may represent a potential example of a pleiotropic gene whose functions extend beyond cardiac tissue. Nevertheless, the presence of *FLNC* in studies of both DCM and ASD should not be interpreted as evidence of a direct clinical relationship between these disorders. Rather, it highlights the possibility that shared molecular pathways may contribute to distinct phenotypes in different organ systems.

Another gene that emerged prominently in our cohort was *MYH6*, which encodes the alpha-heavy chain of cardiac myosin, one of the major contractile proteins of the myocardium. Several studies have demonstrated that pathogenic *MYH6* variants contribute to both dilated and hypertrophic cardiomyopathies [30,40]. Structural alterations affecting highly conserved regions of the protein may impair sarcomeric function and myocardial contractility, ultimately promoting ventricular remodeling and heart failure. More recently, transcriptomic and network-based studies have identified *MYH6* among the key genes associated with heart failure secondary to DCM, further supporting its role in disease pathogenesis [41].

A particularly intriguing observation is that *MYH6* has also been implicated in neurological research. Recent studies have suggested its involvement in biological pathways related to oxidative phosphorylation, mitochondrial respiration, ATP metabolism, and oxidative stress, all of which are relevant to neurodegenerative disorders such as PD [42,43]. Furthermore, *MYH6* has been reported among genes potentially associated with ASD [38,44]. Although these findings do not imply a direct pathogenic link between DCM and neurological disorders, they support the hypothesis that genes involved in fundamental cellular functions may contribute to disease susceptibility across multiple tissues.

The most prominent finding of our study concerns *TTN*, which showed the highest number of variants overall, including both pathogenic variants and variants of uncertain significance (VUS). This observation is fully consistent with the current literature, which identifies *TTN* as the major genetic determinant of DCM [45,46]. Titin is the largest known protein in the human body and serves as a central structural component of the sarcomere, contributing to mechanical stability, elasticity, signal transduction, and force generation [47,48,49].

Truncating variants of TTN (TTNtv) are recognized as the most common genetic cause of DCM and account for approximately 15–25% of familial and advanced forms of the disease [45]. Experimental evidence suggests that TTNtv may impair sarcomere assembly and function, leading to contractile dysfunction, altered cellular signaling, mitochondrial abnormalities, and progressive myocardial remodeling [50]. Environmental and metabolic stressors, including obesity and increased mechanical load, may further amplify the detrimental effects of *TTN* variants and accelerate disease progression [50].

Beyond its established role in cardiomyopathy, *TTN* has also been reported in neurological research. Whole-exome sequencing studies have identified *TTN* among genes associated with ASD [51], while other investigations have suggested interactions between titin-related pathways and proteins implicated in schizophrenia, including DISC1 and AKT1 [52,53,54,55]. Although these observations derive from independent studies and involve different variants, they reinforce the concept that certain genes may contribute to multiple phenotypes through distinct molecular mechanisms.

An additional finding emerging from our analysis is the recurrent involvement of genes such as *RYR2*, *SCN5A*, *RBM20*, *LAMA4*, and others in both DCM and neurological disease literature. *RYR2*, for example, plays a critical role in calcium homeostasis and intracellular signaling, processes that are essential in both cardiomyocytes and neurons. Similarly, *SCN5A* encodes a voltage-gated sodium channel whose dysfunction may affect cellular excitability. Although these overlaps are biologically plausible, their precise clinical and mechanistic significance remains uncertain and requires further investigation.

One of the most striking aspects of our study is the substantial representation of genes previously reported in ASD. Several epidemiological studies have identified increased rates of cardiovascular abnormalities among individuals with ASD, while genetic investigations have suggested the existence of shared molecular pathways influencing both neurodevelopmental and cardiovascular phenotypes [56,57,58]. However, our findings should not be interpreted as evidence that patients with DCM are at increased risk of ASD, or vice versa. Instead, they suggest that selected genes may exert pleiotropic effects across different biological systems.

A similar consideration applies to neurodegenerative disorders such as AD and PD. Increasing evidence indicates that cardiovascular and neurodegenerative diseases share multiple biological pathways, including inflammation, oxidative stress, mitochondrial dysfunction, vascular dysregulation, and altered proteostasis [18]. Moreover, genes such as *PSEN1* and *PSEN2* have been associated with both neurodegenerative and cardiovascular phenotypes [26,27]. Although we did not identify variants in these genes in our cohort, their inclusion among DCM-associated genes further illustrates the complexity of the genetic networks potentially linking cardiac and neurological disorders.

Importantly, the results of the present study should be interpreted within the framework of gene-level overlap rather than phenotype-level overlap. None of the patients included in our cohort had a documented neurological disorder at the time of enrollment, and the variants identified in DCM patients are not necessarily the same variants previously associated with neurological diseases. Consequently, our data do not demonstrate a direct clinical relationship between DCM and neurological disorders. Rather, they indicate that several genes implicated in DCM have also been independently associated with neurological phenotypes, supporting the concept of genetic pleiotropy and shared biological pathways.

From a translational perspective, the identification of genes implicated in both cardiovascular and neurological disorders may contribute to a more integrated understanding of complex disease mechanisms. Although the present findings do not support direct clinical associations, they highlight candidate genes that deserve further investigation in multidisciplinary genomic studies integrating cardiac and neurological phenotyping. In this context, the concept of genetic pleiotropy is increasingly recognized across medical disciplines. Many genes participate in fundamental cellular processes such as cytoskeletal organization, intracellular signaling, mitochondrial function, ion-channel regulation, and protein homeostasis. Consequently, variants affecting these genes may contribute to distinct phenotypes in different organs depending on the specific variant, tissue-specific expression patterns, environmental influences, and modifying genetic factors. Similar observations have been reported in other apparently unrelated disease groups, including cancer and neurodegenerative disorders, where shared molecular pathways and regulatory mechanisms involving non-coding RNAs may contribute to divergent clinical phenotypes [59,60]. Overall, our findings are consistent with this framework and suggest that some DCM-associated genes may exert biological effects extending beyond the cardiovascular system.

The database and literature review identified genes included in the ClinGen DCM curation framework that have also been independently reported in neurological disorders. This descriptive co-annotation does not demonstrate that the same variants are involved in the cardiac and neurological phenotypes, that these genes or variants are enriched in patients with DCM relative to a control population, or that shared pathogenic mechanisms or clinical comorbidity exist. The findings should therefore be considered hypothesis-generating and used to prioritize future controlled studies incorporating standardized neurological phenotyping, variant-level comparisons, and functional validation.

### 4.2. Limitations

Several limitations should be acknowledged. First, this was a single-center study involving a relatively modest sample size, which may limit the generalizability of the findings. Second, no control group was included, preventing comparison of variant frequencies with those observed in healthy individuals or other relevant populations. Because the variant-filtering strategy required a minor allele frequency below 1%, rarity was built into the analytic pipeline and cannot substitute for a control group or demonstrate enrichment in patients with DCM. Third, although no neurological disorders were documented in the available clinical records, systematic neurological, neuropsychological, or instrumental evaluations were not performed. Therefore, the absence of clinically recognized neurological disease does not exclude the presence of subclinical manifestations. Fourth, a large proportion of the identified variants were classified as VUS. Because the interpretation of VUS is inherently dynamic and may change as new evidence becomes available, caution is warranted when drawing biological conclusions from these findings. Fifth, the overlap between DCM-associated genes and neurological disorders was established through HGMD interrogation and literature review rather than through direct phenotypic characterization of the study population. Consequently, the reported associations should be considered exploratory and hypothesis-generating. Finally, no functional studies were performed to validate the biological consequences of the identified variants. Future investigations integrating genomic, transcriptomic, phenotypic, and functional approaches will be necessary to clarify the mechanistic significance of the observed genetic overlaps.

## 5. Conclusions

Our findings are consistent with the prominent role of *TTN*, *FLNC*, and *MYH6* in the genetic landscape of DCM and support that several DCM-associated genes have also been independently reported in neurological disorders. Furthermore, just under 20% are variants likely to be pathogenic or associated with pathogenicity. Importantly, these observations should be interpreted as evidence of gene-level overlap, rather than as proof of a direct clinical association between DCM and neurological disease. Within this framework, the results support the hypothesis that selected DCM-associated genes may exert pleiotropic effects across cardiac and nervous system pathways. Larger multicenter studies integrating detailed cardiovascular and neurological phenotyping, appropriate control groups, and functional validation will be required to clarify the biological and clinical relevance of these shared genetic pathways.

## Figures and Tables

**Table 1:** List of genes differently classified in dilated cardiomyopathy according to Clinical Genome Resource.

Gene	Inheritance	Classification	Gene	Inheritance	Classification
*ABCC9*	AD	Limited	*MYPN*	AD	Moderate
*ACTC1*	AD	Moderate		AD	Limited
*ANKRD1*	AD	Limited	*MYZAP*	AR	Moderate
*BAG3*	AD	Definitive	*NEBL*	AD	Limited
*BAG5*	AR	Moderate	*NEXN*	AD	Strong
*CDH2*	AD	Limited	*NPPA*	AR	No Known Disease Relationship
*CSRP3*	AD	Limited	*NRAP*	AR	Strong
*CTF1*	AD	Limited	*OBSCN*	AD	Limited
*DES*	AD	Definitive	*PDLIM3*	AD	Disputed
*DSG2*	AD	Limited	*PKP2*	AD	Disputed
*DTNA*	AD	Limited	*PLEKHM2*	AR	Moderate
*EYA4*	AD	Limited	*PPA2*	AR	Strong
*FBXO32*	AR	Limited	*PPP1R13L*	AR	Definitive
*FLII*	AR	Moderate	*PRDM16*	AD	Strong
*FLNC*	AD	Definitive	*PSEN1*	AD	Disputed
*GATA6*	AD	Limited	*PSEN2*	AD	Limited
*GATAD1*	AR	Limited	*RBM20*	AD	Definitive
*GET3*	AR	Limited	*RPL3L*	AR	Moderate
*ILK*	AD	Limited	*RTKN2*	AR	No Known Disease Relationship
*JPH2*	AR	Strong	*RYR2*	AD	Limited
	AD	Limited	*SCN5A*	AD	Definitive
*LAMA4*	AD	Limited	*SGCD*	AD	Limited
*LDB3*	AR	Strong	*TBX20*	AD	Strong
	AD	Limited	*TCAP*	AD	Limited
*LRRC10*	AR	No Known Disease Relationship	*TMOD1*	AR	Limited
*LMNA*	AD	Definitive	*TNNC1*	AD	Definitive
*LMOD2*	AR	Definitive	*TNNI3*	AD, AR	Strong
*MIB1*	AD	Limited	*TNNI3K*	AD	Moderate
*MYBPC3*	AD, AR	Limited	*TNNT2*	AD	Definitive
*MYH6*	AD	Limited	*TPM1*	AD	Moderate
*MYH7*	AD	Definitive	*TTN*	AD	Definitive
*MYL2*	AD	Limited	*VCL*	AD	Strong
*MYL3*	AD	Disputed	*VEZF1*	AD	No Known Disease Relationship
*MYLK3*	AD	Moderate			

ClinGen is primarily funded by the National Human Genome Research Institute (NHGRI). The following information was obtained from the Clinical Genome Resource (http://www.clinicalgenome.org (accessed on 17 April 2026)). The updated classifications are at http://www.clinicalgenome.org [29]. **Legend**: AD: Autosomal Dominant; AR: Autosomal Recessive; *ABCC9*: ATP-BINDING CASSETTE, SUBFAMILY C, MEMBER 9; *ACTC1*: ACTIN, ALPHA, CARDIAC MUSCLE; *ANKRD1*: ANKYRIN REPEAT DOMAIN-CONTAINING PROTEIN 1; *BAG3*: BAG COCHAPERONE 3; *BAG5*: BAG COCHAPERONE 5; *CDH2*: CADHERIN 2; *CSRP3*: CYSTEINE- AND GLYCINE-RICH PROTEIN 3; *CTF1*: CARDIOTROPHIN 1; *DES*: DESMIN; *DSG2*: DESMOGLEIN 2; *DTNA*: DYSTROBREVIN, ALPHA; *EYA4*: EYA TRANSCRIPTIONAL COACTIVATOR AND PHOSPHATASE 4; *FBXO32*: F-BOX ONLY PROTEIN 32; *FLII*: FLII ACTIN REMODELING PROTEIN; *FLNC*: FILAMIN C; GATA6: GATA-BINDING PROTEIN 6; *GATAD1*: GATA ZINC FINGER DOMAIN-CONTAINING PROTEIN 1; *GET3*: GUIDED ENTRY OF TAIL-ANCHORED PROTEINS FACTOR 3, ATPase; *ILK*: INTEGRIN-LINKED KINASE; *JPH2*: JUNCTOPHILIN 2; *LAMA4*: LAMININ, ALPHA-4; *LDB3*: LIM DOMAIN-BINDING 3; *LRRC10*: LEUCINE-RICH REPEAT-CONTAINING PROTEIN 10; *LMNA*: LAMIN A/C; *LMOD2*: LEIOMODIN 2; *MIB1*: MIB E3 UBIQUITIN PROTEIN LIGASE 1; *MYBPC3*: MYOSIN-BINDING PROTEIN C, CARDIAC; *MYH6*: MYOSIN, HEAVY CHAIN 6, CARDIAC MUSCLE, ALPHA; *MYH7*: MYOSIN, HEAVY CHAIN 7, CARDIAC MUSCLE, BETA; *MYL2*: MYOSIN, LIGHT CHAIN 2, REGULATORY, CARDIAC, SLOW; *MYL3*: MYOSIN, LIGHT CHAIN 3, ALKALI, VENTRICULAR, SKELETAL, SLOW; *MYLK3*: MYOSIN LIGHT CHAIN KINASE 3; *MYPN*: MYOPALLADIN; *MYZAP*: MYOCARDIAL ZONULA ADHERENS PROTEIN; *NEBL*: NEBULETTE; *NEXN*: NEXILIN F-ACTIN-BINDING PROTEIN; *NPPA*: NATRIURETIC PEPTIDE PRECURSOR A; *NRAP*: NEBULIN-RELATED ANCHORING PROTEIN; *OBSCN*: OBSCURIN; *PDLIM3*: PDZ AND LIM DOMAIN PROTEIN 3; *PKP2*: PLAKOPHILIN 2; *PLEKHM2*: PLECKSTRIN HOMOLOGY DOMAIN-CONTAINING PROTEIN, FAMILY M, MEMBER 2; *PPA2*: PYROPHOSPHATASE, INORGANIC, 2; *PPP1R13L*: PROTEIN PHOSPHATASE 1, REGULATORY SUBUNIT 13-LIKE; *PRDM16*: PR DOMAIN-CONTAINING PROTEIN 16; *PSEN1*: PRESENILIN 1; *PSEN2*: PRESENILIN 2; *RBM20*: RNA-BINDING MOTIF PROTEIN 20; *RPL3L*: RIBOSOMAL PROTEIN L3-LIKE; *RYR2*: RYANODINE RECEPTOR 2; *RTKN2*: RHOTEKIN 2; *SCN5A*: SODIUM VOLTAGE-GATED CHANNEL, ALPHA SUBUNIT 5; *SGCD*: SARCOGLYCAN, DELTA; *TBX20*: T-BOX TRANSCRIPTION FACTOR 20; *TCAP*: TITIN-CAP; *TMOD1*: TROPOMODULIN 1; *TNNC1*: TROPONIN C, SLOW; *TNNI3*: TROPONIN I, CARDIAC; *TNNI3K*: TNNI3-INTERACTING KINASE; *TNNT2*: TROPONIN T2, CARDIAC; *TPM1*: TROPOMYOSIN 1; *VCL*: VINCULIN; *VEZF1:* VASCULAR ENDOTHELIAL ZINC FINGER 1.

## Data Availability

The original data presented in the current study are openly available at the European Nucleotifde Archive (ENA) under the accession number PRJEB114382.

## Supplementary Materials

The following supporting information can be downloaded at: https://www.mdpi.com/article/10.3390/diagnostics16152395/s1, Supplementary Table S1: List of the 211 cardiomyopathy genes used for in silico filtering of the exome-sequencing data; Supplementary Table S2: List of the 105 cases of dilated cardiomyopathy analyzed. The table presents the demographic data (age and sex) and the genetic profile of the subjects, specifying the gene involved and the corresponding variant; Supplementary Table S3: List of the 64 genes validated for DCM. For each gene, the following information is provided: the reference sequence (Ref. Seq.) number listed in the HGMD database; the number of genetic variants identified in our study cohort; the disease/clinical phenotype associated with CNS disorders; and the relevant bibliographic references cited by HGMD.
