# Supplementary material for: Genetic Overlap Between Dilated Cardiomyopathy and Neurological Disorders: Insights from a Next-Generation Sequencing Study
Source: Diagnostics (Basel). 2026 Jul 30;16(15):2395. doi: 10.3390/diagnostics16152395 (PMC13465321; doi:10.3390/diagnostics16152395)
Supplement: Supplementary file 1 [file diagnostics-16-02395-s001.zip › Supplementary Table S1.pdf]

Supplementary Table S1. List of the 211 cardiomyopathy genes used for in silico filtering of the exome-sequencing data

**GENE**	ABCC9, ABCD4, ABCG5, ABCG8, ACSF3, ACTA2, ACTC1, ACTN2, ACVRL1, AGK, AKAP9, ALMS, ANK2, ANKRD, APOB, APOE, ARNTL, ARNTL2, BAG3, BHLHE40, BHLHE4, BMPR2, BRAF, CACNA1C, CACNA2D1, CACNB2, CALM1, CALM2, CALM3, CAP2, CARMIL3, CASQ2, CAV3, CAVIN1, CAVIN4, CD320, CLOCK, COA3, COA5, COL3A1, COL5A1, COL5A2, COQ7, COQ8A, COX14, COX20, CRY1, CRY2, CRYAB, CSNK1E, CSNK2A1, CSRP3, CTNNA3, DES, DLG4, DMD, DNAJC19, DPP6, DSC2, DSG2, DSP, DTNA, EFEMP1, ELAC2, EMILIN1, ENG, EYA4, FBN1, FBN2, FBXL21P, FBXL3, FGF12, FKTN, FLNA, FLNC, FOXE3, FXN, GATD1, GJA5, GLA, GPD1L, GTPBP3, HAND1, HAND2, HCN4, HFE, HRAS, ILK, JPH2, JUP, KCNA5, KCND2, KCND3, KCNE1, KCNE2, KCNE3, KCNE5, KCNH2, KCNJ2, KCNJ5, KCNJ8, KCNQ1, KLHL24, KRAS, LAMA4, LAMP2, LDB3, LDLR, LIPA, LMBRD1, LMNA, LOX, LRP6, LTBP3, MCEE, MED12, MFAP5, MIB1, MLYCD, MMAA, MMAB, MMACHC, MMADHC, MMUT, MRPL44, MTRR, MYBPC3, MYH11, MYH6, MYH7, MYL2, MYL3, MYLK, MYLK2, MYOZ2, MYPN, MYZAP, NDUFAF5, NEBL, NEXN, NFIL3, NNT, NPPA, NR1D1, NR1D2, NUP155, OBSL1, PCCA, PCCB, PCSK9, PER1, PER2, PER3, PKP2, PLN, PLOD1, PPP1R13L, PRDM16, PRKAG1, PRKAG2, PSEN1, PSEN2, PTPN11, RAF1, RANGRF, RBM20, RORA, RORB, RORC, RYR2, SCN10A, SCN1B, SCN2B, SCN3B, SCN4B, SCN5A, SDHA, SDHD, SEMA3A, SGCD, SHOX2, SLC19A2, SLC25A4, SLC2A10, SLMAP, SMAD3, SMAD4, SNTA1, SOS1, SPHK1, SUCLA2, SUCLG1, TAZ, TCAP, TGFB2, TGFB3, TGFBR1, TGFBR2, TGFBR3, THSD4, TIMELESS, TMEM43, TMPO, TNNC1, TNNI3, TNNT2, TNXB, TPM1, TRPM4, TTN, TTR,VCL
