# Supplementary material for: Genetic Overlap Between Dilated Cardiomyopathy and Neurological Disorders: Insights from a Next-Generation Sequencing Study
Source: Diagnostics (Basel). 2026 Jul 30;16(15):2395. doi: 10.3390/diagnostics16152395 (PMC13465321; doi:10.3390/diagnostics16152395)
Supplement: Supplementary file 1 [file diagnostics-16-02395-s001.zip › Supplementary Table S2.pdf]

Supplementary Table S2. List of the 105 cases of DCM analyzed. The table presents the demographic data (age and sex) and the genetic profile of the subjects, specifying the gene involved and the corresponding variant

ID	Age	Sex	Gene	Coding Sequence	Protein
DCM1	30	F	NEXN	c.1419_1421delAAG	p.Arg475del
			TTN	c.77290G>A	p.Gly25764Arg
			TTN	c.63578G>A	p.Arg21193His
DCM2	67	F	TTN	c.104564C>A	p.Ser34855Tyr
DCM4	34	F	TTN	c.98431C>T	p.Arg32811Cys
			PKP2	c.586C>T	p.Arg196Cys
DCM5	60	M	TTN	c.47041C>A	p.Pro15684Thr
			SCN5A	c.*1932C>T	3'UTR
DCM6	59	M	MYZAP	c.640G>T	p.Glu214*
DCM7	36	F	MIB1	c.2827G>T	p.Val943Phe
			TTN	c.85847G>A	p.Arg28616Gln
DCM8	58	F	LAMA4	c.673G>A	p.Ala225Thr
DCM10	53	M	MYH6	c.2368A>G	p.Met790Val
			NEBL	c.1316G>A	p.Arg439Gln
DCM12	62	F	DES	c.937G>A	p.Ala313Thr
			TTN	c.60684_60687delGACA	p.Thr20229Serfs*2
DCM13	38	M	MYBPC3	c.1291G>A	p.Asp431Asn
DCM15	56	F	TTN	c.100585T>C	p.Trp33529Arg
			TTN	c.56806C>T	p.Arg18936Ter
DCM16	53	M	FLNC	c.917G>A	p.Gly306Asp
DCM18	38	M	MYH6	c.4533C>G	p.Ile1511Met
			JPH2	c.753C>G	p.Ser251Arg
DCM19	45	M	MYH6	c.2258T>C	p.Ile753Thr
			ANKRD1	c.3G>T	p.Met1Ile
DCM20	56	M	TPM1	c.64G>A	p.Ala22Thr
			TTN	c.94453G>A	p.Ala31485Thr
DCM21	23	M	TTN	c.170C>T	p.Ser57Phe
			LAMA4	c.3034T>C	p.Tyr1012His
DCM22	63	F	FLNC	c.146A>C	p.His49Pro
			PRDM16	c.3724G>A	p.Glu1242Lys
DCM23	67	F	FLNC	c.146A>C	p.His49Pro
			PRDM16	c.3724G>A	p.Glu1242Lys
DCM24	49	F	TTN	c.44915T>G	p.Val14972Gly
			TTN	c.42109C>T	p.Gln1403Ter
			JPH2	c.1149G>T	p.Lys383Asn
DCM25	44	M	TTN	c.99254G>A	p.Arg33085His
			RBM20	c.613C>A	p.Gln205Lys
DCM26	49	M	TTN	c.84976C>T	p.Arg28326Trp
DCM27	71	M	TTN	c.100585T>C	p.Trp33529Arg
DCM28	58	M	SCN5A	c.3374C>G	p.Ala1125Gly
DCM30	50	M	TTN	c.50812G>C	p.Glu16938Gln
DCM31	76	M	RBM20	c.3572A>G	p.Gln1191Arg
DCM36	55	M	TTN	c.917C>G	p.Ser306Cys
			MYH7	c.3133C>T	p.Arg1045Cys
			RMB20	c.3655G>A	p.Val1219Met

DCM37	28	M	NEXN	c.1112C>T	p.Pro371Leu
			NEXN	c.1958T>C	p.Met653Thr
DCM38	50	M	TTN	c.5582G>A	p.Arg1861His
			ILK	c.7G>C	p.Asp3His
DCM39	56	M	TTN	c.95415C>A	p.Phe31805Leu
DCM41	45	M	RYR2	c.12745A>G	p.Arg4249Gly
			FLNC	c.5407C>T	p.Arg1803Trp
DCM42	79	M	JPH2	c.1149G>T	p.Lys383Asn
			MYBPC3	c.2834G>T	p.Arg945Leu
DCM43	57	M	FLNC	c.2672C>T	p.Thr891Met
DCM45	63	F	PRDM16	c.427T>A	p.Cys143Ser
DCM46	36	F	JPH2	c.1715G>A	p.Arg572His
DCM51	56	F	RBM20	c.5T>A	p.Val2Glu
			DTNA	c.1571G>A	p.Arg524His
DCM52	74	M	MIB1	c.2026C>T	p.Arg676Ter
			TTN	c.58784C>T	p.Ser19595Phe
			TTN	c.29638G>A	p.Glu9880Lys
DCM53	49	M	TTN	c.15605T>C	p.Met5202Thr
			MYH6	c.1042G>A	p.Ala348Thr
DCM54	25	M	TTN	c.34855+1G>A	Loss of Function
DCM56	23	F	TTN	c.5824G>C	p.Ala1942Pro
DCM57	44	F	TTN	c.105424G>A	p.Glu35142Lys
DCM58	31	M	RBM20	c.2761A>T	p.Ile921Phe
DCM60	74	M	TTN	c.72556A>G	p.Lys24186Glu
			TTN	c.27030T>A	p.Ser9010Arg
DCM61	52	M	TTN	c.24571G>A	p.Glu8191Lys
			NEXN	c.1419_1424delAAG	p.Arg475del
DCM62	33	M	TTN	c.7401G>T	p.Lys2467Asn
			TTN	c.77290GZA	p.Gly25764Arg
DCM63	56	M	EYA4	c.155A>C	p.Lys52Thr
			JPH2	c.753C>G	p.Ser251Arg
DCM64	61	M	TTN	c.87260_87264delCAACC	p.Ala29087Aspfs*4
DCM66	63	M	BAG3	c.826C>T	p.Arg276Trp
DCM67	60	F	MYH7	c.323G>A	p.Arg108His
DCM69	69	M	SCN5A	c.1398G>T	p.Leu466Phe
DCM72	60	F	FLNC	c.146A>C	p.His49Pro
			MYH6	c.3391A>G	p.Arg1131Gly
DCM74	63	M	FLNC	c.6854C>A	p.Pro2285His
			TTN	c.87724T>C	p.Ser29242Pro
DCM77	58	F	MYH7	c.4690G>A	p.Glu1564Lys
DCM78	33	F	MYH7	c.4690G>A	p.Glu1564Lys
			TNNI3	c.220C>G	p.Arg74Gly
DCM80	76	M	FLNC	c.1309C>T	p.Arg437Cys
DCM85	43	F	FLNC	c.874G>A	p.Val292Met
DCM86	61	F	RYR2	c.5978G>A	p.Arg1993His
			SGCD	c.190A>G	p.Ile64Val
DCM87	50	F	FLNC	c.5504A>C	p.Gln1835Pro
DCM88	21	M	MYH6	c.2842G>A	p.Glu948Lys
DCM89	27	F	TTN	c.1453A>G	p.Lys485Glu
			PKP2	c.2028G>T	p.Trp676Cys
DCM90	62	F	MYBPC3	c.2518G>A	p.Val840Met

DCM91	42	F	TTN	c.104413C>T	p.Arg34805Ter
DCM92	54	M	MYBPC3	c.557C>T	p.Pro186Leu
DCM93	63	M	DES	c.407T>A	p.Leu136His
DCM94	56	F	TTN	c.63380G>A	p.Arg21127His
DCM97	63	M	LMNA	c.569G>A	p.Arg190Gln
DCM98	60	M	TTN	c.75539G>T	p.Gly25180Val
DCM100	65	F	SCN5A	c.2924G>A	p.Arg975Gln
DCM101	19	M	NEXN	c.344A>G	p.Glu115Gly
DCM102	64	M	TTN	c.9577C>T	p.Arg3193Ter
			ABCC9	c.430G>A	p.Ala144Thr
DCM103	38	M	TTN	c.29257_29258delGA	p.Glu9753Serfs*6
DCM104	78	M	TTN	c.66592delG	p.Ala22198Leufs*7
DCM105	56	M	MYBPC3	c.122G>A	p.Arg41His
			RBM20	c.1094G>T	p.Gly365Val
DCM106	54	F	MYL2	c.275-2A>G	Loss of Function
			MYH6	c.2794G>A	p.Glu932Lys
DCM107	68	M	DES	c.937G>A	p.Ala313Thr
DCM108	34	F	PKP2	c.1742G>A	p.Gly581Glu
DCM111	59	M	FLNC	c.3180delT	p.Asp1061Ilefs*17
DCM112	48	F	TTN	c.87367A>C	p.Ser29123Arg
			DTNA	c.473C>G	p.Ser158Cys
DCM113	64	M	TTN	c.95565_95566dupAC	p.Arg31856Hisfs*14
DCM114	25	M	SCN5A	c.3911C>T	p.Thr1304Met
DCM115	74	M	MYBPC3	c.1828G>C	p.Asp610His
			DTNA	c.1571G>A	p.Arg524His
DCM116	28	M	TTN	c.102826C>T	p.Arg34276Trp
DCM118	58	M	MYH6	c.3988G>A	p.Ala1330Thr
DCM119	68	M	SCN5A	c.4534C>T	p.Arg1512Trp
DCM122	59	M	TTN	c.68210C>T	p.Ala22737Val
DCM123	51	M	TTN	c.83821G>A	p.Glu27941Lys
			MYH6	c.5485G>C	p.Glu1829Gln
DCM127	30	M	RBM20	c.326C>T	p.Ala109Val
DCM128	70	M	LMNA	c.569G>A	p.Arg190Gln
			FLNC	c.4057G>A	p.Val13531Ile
			RBM20	c.1900C>T	p.Arg634Trp
DCM131	44	M	TTN	c.89684C>T	p.Ser29895Leu
			DTNA	c.473C>G	p.Ser158Cys
DCM132	61	M	FLNC	c.146A>C	p.His49Pro
DCM134	56	F	TTN	c.73926T>G	p.His24642Gln
DCM135	58	M	TTN	c.98075C>G	p.Thr32692Arg
DCM136	71	F	DES	c.407T>A	p.Leu136His
			TTN	c.105562A>C	p.Ile35188Leu
DCM137	54	F	TTN	c.60314T>G	p.Val20105Gly
			MYPN	c.1019C>G	p.Ser340Cys
DCM138	47	M	TTN	c.100585T>C	p.Trp33529Arg
DCM139	65	M	TTN	c.60242A>G	p.Asp20081Gly
DCM140	70	M	TTN	c.16546G>T	p.Asp5516Tyr
DCM141	62	M	TPM1	c.563+6C>G	Splice Region
DCM142	58	M	PRDM16	c.934G>T	p.Asp312Tyr
			TTN	c.102428T>C	p.Met34143Thr

DCM143	36	M	TNNT2	c.793C>T	p.Arg265Ter
			RYR2	c.8477T>C	p.Ile2826Thr
DCM144	52	M	TTN	c.55972C>T	p.Arg18658Ter
DCM145	69	M	TTN	c.7426G>C	p.Val2476Leu
DCM146	43	M	TTN	c.105590G>A	p.Gly35197Asp
DCM147	77	M	MYBPC3	c.2915G>A	p.Arg972Gln
DCM148	37	M	EYA4	c.878A>G	p.Tyr293Cys
DCM149	41	M	PRDM16	c.2062G>A	p.Ala688Thr

Note Well: All genetic variants shown in blue are classified by the ACGM as Variants of Uncertain Significance (VUS), while those shown in orange are classified as Likely Pathogenic variants and those in red are classified as Pathogenic.
