# Supplementary material for: Genetic Overlap Between Dilated Cardiomyopathy and Neurological Disorders: Insights from a Next-Generation Sequencing Study
Source: Diagnostics (Basel). 2026 Jul 30;16(15):2395. doi: 10.3390/diagnostics16152395 (PMC13465321; doi:10.3390/diagnostics16152395)
Supplement: Supplementary file 1 [file diagnostics-16-02395-s001.zip › Supplementary Table S3.pdf]

Supplementary Table S3. List of the 64 genes validated for DCM. For each gene, the following information is provided: the reference sequence (Ref.Seq.) number listed in the HGMD database; the number of genetic variants identified in our study cohort; the disease/clinical phenotype associated with CNS disorders; and the relevant bibliographic references cited by HGMD

Gene	Ref.Seq. (HGMD)	Genetic Variants Identified	CSN Diseases	Reference
ABCC9	NM_005691.4	1	ASD	PMID: 35982159; PMID: 35982160
ACTC1	NM_005159.5	\	ASD	PMID: 28714951; PMID: 31785789
ANKRD1	NM_014391.3	1	ASD	PMID: 35982159; PMID: 35982160
BAG3	NM_004281.4	1	ASD	PMID: 35982159; PMID: 31785789
BAG5	NM_004873.4	\	ASD	PMID: 35982159; PMID: 35982160
CDH2	NM_001792.5	\	ASD	PMID: 35982159; PMID: 28867142; PMID: 29346770
			AD	PMID: 23752245
CSRP3	NM_003476.5	\	No CSN Disease	
CTF1	NM_001330.5	\	No CSN Disease	
DES	NM_001927.4	2	ASD	PMID: 35982159
DSG2	NM_001943.5	\	ASD	PMID: 35982159; PMID: 35982160
DTNA	NM_001390.5	2	ASD	PMID: 35982159; PMID: 35982160; PMID: 28191890
EYA4	NM_004100.5	2	ASD	PMID: 35982160; PMID: 35982159
FBXO32	NM_058229.4	\	ASD	PMID: 35982159
FLII	NM_001256264.2	\	ASD	PMID: 35982160; PMID: 35982159
FLNC	NM_001458.5	9	ASD	PMID: 35982160; PMID: 35982160; PMID: 34312540
			FTD	PMID: 26555887
GATA6	NM_005257.6	\	ASD	PMID: 35982160; PMID: 35982159
GATAD1	NM_021167.5	\	ASD	PMID: 35982159
GET3	NM_004317.4	\	ASD	PMID: 35982160; PMID: 35982159
ILK	NM_004517.4	1	ASD	PMID: 35982159
JPH2	NM_020433.5	3	ASD	PMID: 35982160
LAMA4	NM_002290.5	2	ASD	PMID: 25363768; PMID: 28714951; PMID: 34011629; PMID: 35982160; PMID: 35982159
LDB3	NM_001080116.1	\	SMA	PMID: 32721234
LRRC10	NM_201550.4	\	ASD	PMID: 35982159
LMNA	NM_170707.4	1	ASD	PMID: 35982160; PMID: 35982159
LMOD2	NM_207163.3	\	ASD	PMID: 35982159
MIB1	NM_020774.4	2	ASD	PMID: 35982160; PMID: 35982159; PMID: 36368308; PMID: 34312540
MYBPC3	NM_000256.3	7	ASD	PMID: 35982159
MYH6	NM_002471.4	9	ASD	PMID: 35982160; PMID: 35982159
MYH7	NM_000257.4	3	ASD	PMID: 35982159
MYL2	NM_000432.4	1	\	\
MYL3	NM_000258.3	\	\	\
MYLK3	NM_182493.3	\	ASD	PMID: 35982160; PMID: 35982159; PMID: 31133750
MYPN	NM_032578.4	1	ASD	PMID: 35982160; PMID: 35982159; PMID: 28867142
MYZAP	NM_001018100.5	1	ASD	PMID: 35982160; PMID: 35982159

NEBL	NM_006393.3	1	ASD	PMID: 35982159
NEXN	NM_144573.4	4	ASD	PMID: 35982160; PMID: 35982160
NPPA	NM_006172.4	\	ASD	PMID: 35982159
NRAP	NM_001261463.2	\	ASD	PMID: 35982160; PMID: 35982159; PMID: 25363768; PMID: 28714951
			ASD	PMID: 25363768; PMID: 28714951; PMID: 34011629; PMID: 35982159; PMID: 35051175
OBSCN	NM_052843.4	\	AD	PMID: 30924900
			Schizophrenia	PMID: 24463507
PDLIM3	NM_014476.6	\	ASD	PMID: 35982160; PMID: 35982159
PKP2	NM_004572.4	3	ASD	PMID: 35982159
PLEKHM2	NM_015164.4	\	ASD	PMID: 35982159
PPA2	NM_176869.3	\	ASD	PMID: 35982159
PPP1R13L	NM_006663.4	\	ASD	PMID: 35982159
PRDM16	NM_022114.4	4	ASD	PMID: 35982159; PMID: 31133750; PMID: 35982160
			AD	PMID: 30021643; PMID: 31914229; PMID: 34102969; PMID: 36133075; PMID: 32917274; PMID: 28350801; PMID: 28532646; PMID: 31282415; PMID: 11796781; PMID: 34679393; PMID: 39903689; PMID: 40016812; PMID: 10441572; PMID: 22503161; PMID: 9831473; PMID: 28008242; PMID: 20634584; PMID: 39024526; PMID: 35065037; PMID: 27743520; PMID: 19797784; PMID: 29091718; PMID: 39713882; PMID: 10655540; PMID: 16941492; PMID: 26350633; PMID: 7596406; PMID: 27785004; PMID: 35850649; PMID: 31920735; PMID: 32477171; PMID: 31182772; PMID: 25104557; PMID: 27930341
PSEN1	NM_000021.4	\	FTD	PMID: 30279455; PMID: 33203472; PMID: 11094121; PMID: 30528349; PMID: 31914217; PMID: 15122701; PMID: 16546171; PMID: 32843152; PMID: 36570531; PMID: 11997713
			Dementia	PMID: 35906014; PMID: 34389718; PMID: 25333068; PMID: 30090657; PMID: 31836585; PMID: 23998997; PMID: 22503161; PMID: 30279455; PMID: 37330543;
			Dementia with Lewy bodies	PMID: 22118943; PMID: 27312774; PMID: 36114914; PMID: 33769990; PMID: 31996268; PMID: 26242991
			PD	PMID: 37198191; PMID: 35861376; PMID: 38397244;
			ASD	PMID: 28191890; PMID: 34011629; PMID: 22495309
			Creutzfeldt-Jakob disease	PMID: 10896268
			Parkinsonism	PMID: 36825052; PMID: 34843019; PMID: 29316780

PSEN2	NM_000447.3	\	AD	PMID: 30822634; PMID: 33769986; PMID: 25937274; PMID: 28243073; PMID: 18667258; PMID: 22312439; PMID: 22312439; PMID: 36133075; PMID: 28106563; PMID: 34102969; PMID: 39903689; PMID: 28350801; PMID: 26422362; PMID: 14623725; PMID: 18350357; PMID: 30954774; PMID: 7651536; PMID: 35120450; PMID: 35491795; PMID: 10732806; PMID: 25323700; PMID: 33855622; PMID: 23383383; PMID: 31914229; PMID: 24885952; PMID: 40016812; PMID: 30119059; PMID: 14769392; PMID: 26522186; PMID: 23990795; PMID: 15258222; PMID: 24559647; PMID: 12433263; PMID: 20375137
			FTD	PMID: 22459598; PMID: 30054184; PMID: 19768372; PMID: 25323700; PMID: 39200345
			PD	PMID: 22118943; PMID: 35861376
			Parkinsonism	PMID: 33601107
			Dementia	PMID: 15130954; PMID: 34720994; PMID: 30279455; PMID: 25333068; PMID: 32317127; PMID: 14681895; PMID: 10631141; PMID: 22503161; PMID: 29525180; PMID: 30090657; PMID: 34389718; PMID: 18727676; PMID: 33061333
			ASD	PMID: 31981491; PMID: 27824329; PMID: 35982159
			Dementia with Lewy bodies	PMID: 18667258; PMID: 22118943; PMID: 26836416; PMID: 21409510
			SLA	PMID: 29525178
			Schizophrenia	PMID: 19232479; PMID: 37517367
RBM20	NM_001134363.3	8	ASD	PMID: 35982159; PMID: 35982160
RPL3L	NM_005061.3	\	AD	PMID: 39713882
			ASD	PMID: 35982159; PMID: 35982160
RTKN2	NM_145307.4	\	ASD	PMID: 35982159
RYR2	NM_001035.3	3	ASD	PMID: 35982159; PMID: 25363768; PMID: 28714951; PMID: 34011629; PMID: 28191890; PMID: 34312540; PMID: 26742492; PMID: 37393044; PMID: 31038196; PMID: 38256266; PMID: 34712263
			Schizophrenia	PMID: 36863698; PMID: 33526774; PMID: 28750581;
			Epilepsy	PMID: 34160719; PMID: 34489640; PMID: 36340715; PMID: 38277927
SCN5A	NM_198056.3	6	ASD	PMID: 35982159; PMID: 34011629; PMID: 36199823; PMID: 28191890
			Epilepsy	PMID: 31440721
SGCD	NM_000337.6	1	ASD	PMID: 35982160; PMID: 35982159
TBX20	NM_001077653.2	\	ASD	PMID: 35982160; PMID: 35982159
TCAP	NM_003673.4	\	ASD	PMID: 35982159
TMOD1	NM_003275.4	\	ASD	PMID: 35982159

TNNC1	NM_003280.3	\	ASD	PMID: 35982159
TNNI3	NM_000363.5	1	ASD	PMID: 35982159
TNNI3K	NM_015978.3	\	ASD	PMID: 35982160; PMID: 35982159
TNNT2	NM_001001430.3	1	ASD	PMID: 35982159
TPM1	NM_001018005.2	2	ASD	PMID: 35982159
TTN	NM_133378.4	54	ASD	PMID: 25363768; PMID: 28714951; PMID: 34011629; PMID: 35982159; PMID: 35982160; PMID: 31133750; PMID: 29327328
			AD	PMID: 30924900
			Schizophrenia	PMID: 26666178; PMID: 38398753
VCL	NM_014000.3	\	ASD	PMID: 35982159; PMID: 35982160
			Epilepsy	PMID: 29619247
VEZF1	NM_007146.3	\	ASD	PMID: 35982159; PMID: 31981491; PMID: 36368308
